# Bioaccumulation of Heavy Metals in Water, Sediments, and Tissues and Their Histopathological Effects on* Anodonta cygnea* (Linea, 1876) in Kabul River, Khyber Pakhtunkhwa, Pakistan

**DOI:** 10.1155/2018/1910274

**Published:** 2018-03-06

**Authors:** Muhammad Iftikhar Khan, Muhammad Khisroon, Ajmal Khan, Naila Gulfam, Muhammad Siraj, Farrah Zaidi, Ahmadullah, Abidullah, Syeda Hira Fatima, Shumaila Noreen, Hamidullah, Zafar Ali Shah, Fazli Qadir

**Affiliations:** ^1^Department of Zoology, University of Peshawar, Khyber Pakhtunkhwa, Pakistan; ^2^Department of Space Science, Institute of Space Technology, Islamabad, Pakistan; ^3^Department of Chemistry, University of Swabi, Khyber Pakhtunkhwa, Pakistan; ^4^Preston University, Islamabad, Pakistan

## Abstract

The present investigation aimed to assess the concentrations of selected heavy metals in water and sediments and their bioaccumulation in tissues of freshwater mussels and their histopathological effects on the digestive gland, gills, and gonads of* Anodonta cygnea*. Water, sediments, and freshwater mussel samples were collected at four sites, that is, reference and polluted sites, along the Kabul River, Khyber Pakhtunkhwa. The polluted sites were receiving effluents from the industrial, agricultural, municipal, and domestic sources. The order of metals in the water was Zn > Pb > Ni > Cu > Mn > Fe > Cr > Cd, in sediments the order was Fe > Zn > Cr > Ni > Mn > Pb > Cu > Cd, and in the soft tissues the order was Fe > Zn > Mn > Pb > Cu > Cr > Ni > Cd. Histopathological alterations observed in polluted sites of Kabul River were inflammation, hydropic vacuolation, and lipofuscin pigments (in digestive gland), gill lamellar fusion, dilated hemolymphatic sinus, clumping, and generation of cilia and hemocytic infiltration (in gills), and atresia, necrosis, granulocytoma, hemocytic infiltration, and lipofuscin pigments (in gonads). The histopathological alterations in the organs of* Anodonta cygnea *can be considered as reliable biomarkers in biomonitoring of heavy metal pollution in aquatic ecosystems.

## 1. Introduction

Advancement in human lifestyle due to science and technology causes contamination of environment. Heavy metals are one of such pollutants that may come from both natural and human activity and could be a serious problem/threat because of their toxicity, long persistence, and bioaccumulation and biomagnification of metals in the food chain [1, 2]. In order to assess the extent of heavy metal toxicity, it is essential to understand water quality parameters [3]. The heavy metals can react with various contents of aquatic environment and can associate with various geochemical phases in the sediments [4]. Geochemical speciation and distribution of metals in the defined chemical fraction have also been used in predicting the potential contamination, bioavailability, and mobility [5–7].

Among aquatic biota, freshwater mussels are desirable organisms for biomonitoring purposes [8–10], since these organisms are in direct contact with polluted parts of water and sediments of their habitats can accumulate high levels of heavy metals in soft parts of their bodies [11]. Freshwater mussels are sensitive indicators of chemical pollution due to their filtration activity [8, 12, 13]. In freshwater mussel, mantle and gills have respiratory and feeding functions [14, 15] and significant potential for accumulation of heavy metals and other pollutants [16–18].

The selection of histopathology as proxy of disease and contamination was based on previous researches that are indications for strong relation between pollution and the histopathology of gills, digestive glands, and gonads [19–22]. Furthermore, histopathological biomarkers have been used to monitor parasites that negatively affect bivalve, fisheries, and aquaculture industry in many countries such as Egypt, Korea, Argentina, Japan, and Spain [23–29].

This study investigated heavy metals (Cd, Cr, Cu, Fe Mn, Ni, Pb, and Zn) in water, sediments, and soft tissues and the effect of these metals on the digestive gland, gills, and gonads of freshwater mussels from the Kabul River, Khyber Pakhtunkhwa, Pakistan.

## 2. Materials and Methods

### 2.1. Study Sites

**Figure 1:**
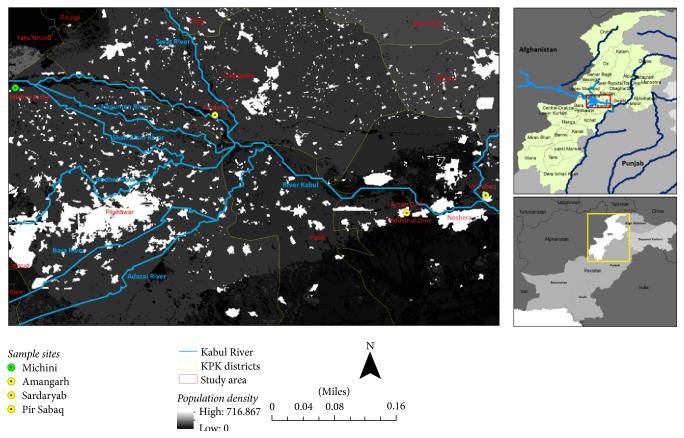
Kabul River and its tributaries. The control site is upstream (green circle), while the experimental sites are downstream (yellow circles). The white patches in the map show high population density regions contributing to high sewage inputs to the river.

Four sites along the stretch of the Kabul River in Khyber Pakhtunkhwa, Pakistan, were selected (Figure 1). The upstream site below Warsak Dam, the Michini Bridge, was designated as the reference/control site (site 1, 34.16851, 71.43245). In the vicinity of this site, human population density is low. Three other downstream sites at Sardaryab (site 2, 34.13338, 71.68944), Amangarh (site 3, 34.00791, 71.93622), and Pir Sabaq (site 4, 34.03035, 72.03796) were designated as polluted/experimental sites. Site 2 is receiving sewage and agricultural inputs from rural areas along the river. Site 3 is receiving sewage inputs from heavily urbanized areas alongside the tributaries such as provincial capital, Peshawar. The same site is also picking up industrial effluents from nearby Amangarh industrial estate. The fourth site is receiving effluents from all these sources and additionally from Nowshera and Mardan cities.

### 2.2. Collection of Water

Representative water samples (*n* = 40) of about 1 liter were collected from the sampling sites in polypropylene 500 ml cleaned bottles. At the sampling time, these bottles were also washed with the respective river water. The samples were collected below the surface about 2-3 feet away from the river banks in such a way that no bubbles were allowed. These water samples were filtered and preserved in 5 ml of 55% HNO_3_ per liter of water to prevent metal adsorption on the inner surface of the container and stored at 4°C before their analyses.

### 2.3. Collection of Sediments

50 g sediment samples (*n* = 10) were collected from a depth of 15 cm from the surface from each site, using a sediment collector with an acid-washed plastic scoop and transported to the laboratory in polyethylene bags. They were dried and passed through a 2 mm sieve.

### 2.4. Collection of Freshwater Mussel Samples

**Figure 2:**
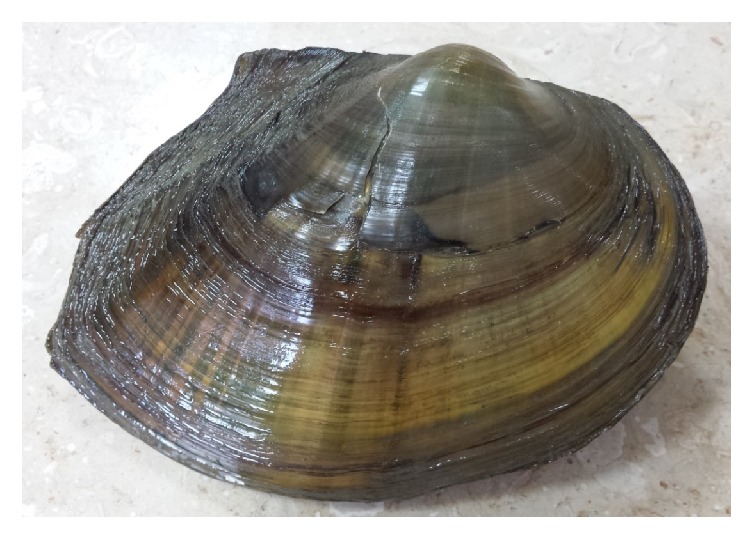
Freshwater mussel* (Anodonta cygnea)*.

Freshwater mussels (*n* = 80) were collected from their natural populations at the four respective sites. The species sampled for the present study was* Anodonta cygnea* (Figure 2). The mussels were collected by hand and placed into clean containers holding water. They were transported to the laboratory, where 40 mussels were kept in 15 L of water for 6 to 7 days. The length, width, and total weight of the mussels were noted (Table 1). Sterilized stainless steel scalpel was used to remove the soft tissue in a laminar flow cabinet lined with a Teflon base. The flesh samples were oven-dried at 60°C and reweighed to determine the dry weight, which is typically only 5–10% of the mussel wet weight, and were depurated overnight in clean and filtered water.

### 2.5. Metal Estimation in Water Samples

100 ml of acidified water samples was evaporated in a volumetric flask on a hot plate and reduced to about 20 ml within a fume cupboard and then a mixture of 5 ml of HNO_3_ (55%) and 10 ml of perchloric acid (70%) was added. The mixture was evaporated on a hot plate until the brown fumes converted into dense white fumes of perchloric acid. The samples were cooled and diluted to 100 ml with double distilled water. The solutions were then analyzed through atomic absorption spectrophotometer (Spectra-AA-700) by using an air acetylene flame for the determination of these metals (Table 2).

### 2.6. Metal Estimation in Sediment Samples

The same procedure was carried out on 5 g of dried sediment samples.

### 2.7. Metal Analysis in Freshwater Mussel Tissues

Metal detection in all the soft tissues was determined by using the method described by Siraj et al. [30]. Briefly, tissues were rinsed with double deionized water and kept on blotting paper. 50 g of each tissue was placed in separate 100 ml volumetric flask. Tissues were digested in 5 ml mixed solution of perchloric and nitric acid. The next day, a fresh mixture of the two acids was added to each tissue. The tissues containing flasks were placed on hot plate and allowed to digest at 200 to 250°C until a transparent and clear solution was obtained. 100 ml double distilled water was added to digested tissues. Heavy metals were analyzed through atomic absorption spectrophotometer (Spectra-AA-700) by using an air acetylene flame for the determination of metals. The dissected samples were fixed in calcium-formaldehyde solution (40% formaldehyde and 10% calcium) and dehydrated in alcohols using ethanol. The mussels were cut at plane so as to include gills, gonads, and digestive gland. Tissues were dehydrated by placing them in 70%, 95%, and finally 100% ethanol. Ethanol from tissues was removed in methyl benzoate solution and then rinsed in benzene and embedded in paraffin. Histological sections (7 *μ*m thick) were cut on a microtome HM335s (Microm GmbH, Bergman, Germany) mounted on slides, dried at 37°C for 24 h, and stored at room temperature until staining. Up to twenty mussels tissue thin sections per site were stained with hematoxylin and eosin and examined for histopathological abnormalities. Sections were studied under light microscope for parasitic infestation assessment and pathological alterations in the target tissues.

### 2.8. Statistical Analysis

One-way analysis of variance (ANOVA) test was used to compare the different sets of data collected from the polluted sites to that of reference site; *P* < 0.05 was considered to show statistical significance, using SPSS v.20.0 software for Windows.

## 3. Results and Discussions

### 3.1. Concentration of Heavy Metals in Water

The results given in Table 2 indicate heavy metal concentrations in water samples collected from sites 1 (reference site), 2, 3, and 4. The concentrations of heavy metals in water samples from both polluted and reference sites were within permissible limits as laid down by NEQS 1997 [31]. However, the respective concentrations of metals showed gradual hike from site 1 to site 4 with metal concentrations for sites 2, 3, and 4 being significantly higher than site 1 (Table 3). The value of zinc was relatively high at the reference site 1 (Table 3), which is understandable as Kabul River originates from Paghman Mountains, so high levels of Zn in headwaters can be associated with release of Zn from various minerals and rocks [32]. With respect to site 1, the levels of zinc were significantly high at the other three sites (Table 2) which can be attributed to addition of domestic and industrial effluents. In Pakistan, these two sources contribute significantly to zinc pollution in freshwater systems [33–35]. Khan et al. [33] found Zn as the second highest metal in Shah Alam River (Ni, Zn, Cu, and Pb) at polluted sites near Peshawar city. A study from Punjab province showed Zn and Cu as the highest metals in River Ravi water at Balloki Headworks [34]. A similar study on heavy metals in the water of River Sutlej at Sulemanki Headworks showed Zn as the second most accumulated metal [35]. Indeed across the globe industrial and sewage discharges are considered the most important sources of surface water pollution [36].

### 3.2. Concentration of Heavy Metals in Sediments

The metals in the sediments were in the order of Fe > Zn > Cr > Ni > Mn > Pb > Cu > Cd. The sediments samples from polluted sites 2, 3, and 4 showed the highest levels of metals, while the reference site demonstrated lowest values (Table 4). In consensus with the present study, Tabinda et al. [34] showed Fe and Zn as the highest metals accumulated in the sediments of River Ravi. Large amount of heavy metals that are bound in sediments due to high surface area and content of humic substances can be released into the water by movements of aquatic organisms [35, 37].

### 3.3. Concentration of Heavy Metals in Soft Tissues of Freshwater Mussels

Bioaccumulation showed peak values in soft tissues of freshwater mussels collected from polluted sites 2–4. The difference was significant when compared to reference site 1 with least pollution (Table 5). The order of these metals in soft tissues of freshwater mussels was Fe > Zn > Mn > Pb > Cu > Cr > Ni > Cd. The higher values are due to low detoxification mechanisms, low metabolic rates, direct exposure of the tissues to metals, and lower rate of elimination of metals. In our study, the degree of metal accumulation in soft tissues followed the same order and spatial variations as found in the associated sediments. Similar results have been presented by a number of studies that demonstrate the sensitivity of mussels to slight changes in heavy metal concentrations in their aquatic habitat [38–40]. Thus, mussel soft tissue can be used to examine environmental conditions over shorter time scales. Heavy metal contents in mussel hemolymph, foot, and mantle tissue can be used as sublethal biomarkers to monitor water quality, stress, or immune responses [18, 19, 41–43]. For several decades, Unionidae family has been used for monitoring pollution status of lakes and rivers [19, 39, 40, 44]. There are a significant number of reports on heavy metals and other elements in adult mussels [45–47] since the Mussel Watch concept was proposed by Goldberg [48]. The selection criteria for being a good pollution indicator do not solely depend on its accumulative index for the particular parameter [45–47]. It also considers other factors such as parasite burden and histological alterations.

### 3.4. Parasitic Infestation

**Figure 3:**
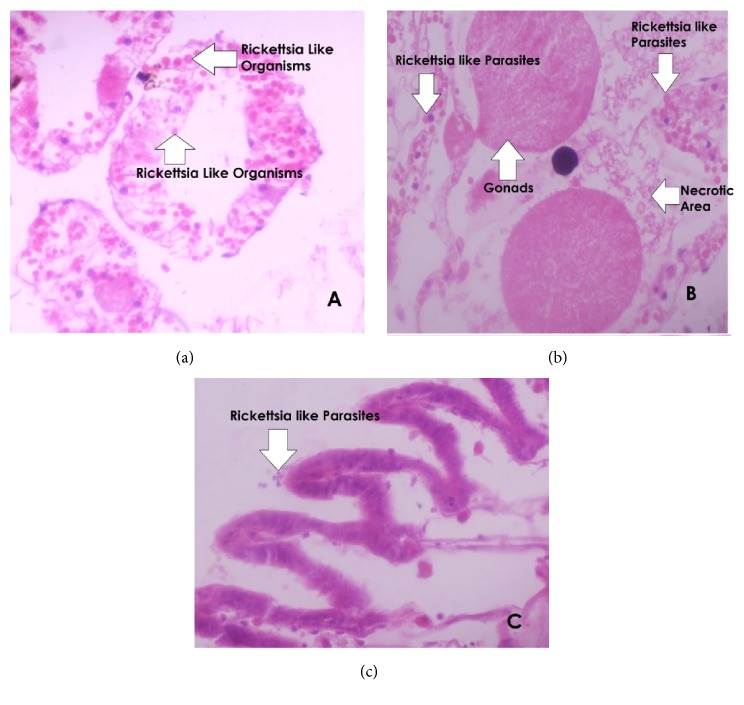
(a) Section of digestive gland showing* Rickettsia*-like organism (×400). (b) Section of gonads showing* Rickettsia*-like organism (×400). (c) Section of gills showing* Rickettsia*-like organism (×400).

The specimens of freshwater mussels were found to be infected with* Rickettsia*-like organisms (RLOs).  Figure 3 shows the density of parasites in the samples from all collection sites. The organ wise density was digestive gland > gills > gonads. The RLOs are intracellular bacterial parasites, known to be common in invertebrates, mussels in particular. The population of freshwater mussels was severely infected with RLOs at downstream sites of Amangarh and Pir Sabaq. Amangarh, in addition to pollution by industrial and domestic effluents, is grossly contaminated [31]. Hence, severe RLOs infection rate is found in the mussels samples collected from Amangarh as compared to other sites.

### 3.5. Histological Study

#### 3.5.1. Digestive Glands

**Figure 4:**
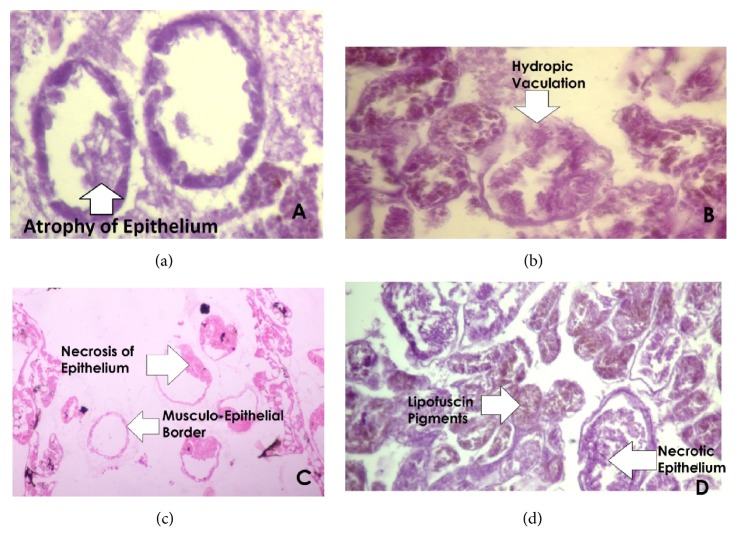
Section of digestive gland showing (a) atrophy of epithelium (×400), (b) hydropic vacuolation (×400), (c) necrosis of epithelium (×400), and (d) lipofuscin pigments (×400).

Histopathological alterations found in digestive gland were atrophy, hydropic vacuolation, necrosis, and lipofuscin. All four types of lesions were present in specimens from all the collection sites. Their percentage wise distribution is shown in Figure 4. Samples from Amangarh possessed 68% atrophy, while those of Pir Sabaq showed 72% lipofuscin pigments. In other studies, high vacuolation in digestive tubules has been found in clam and mussels [49, 50].

#### 3.5.2. Gills

**Figure 5:**
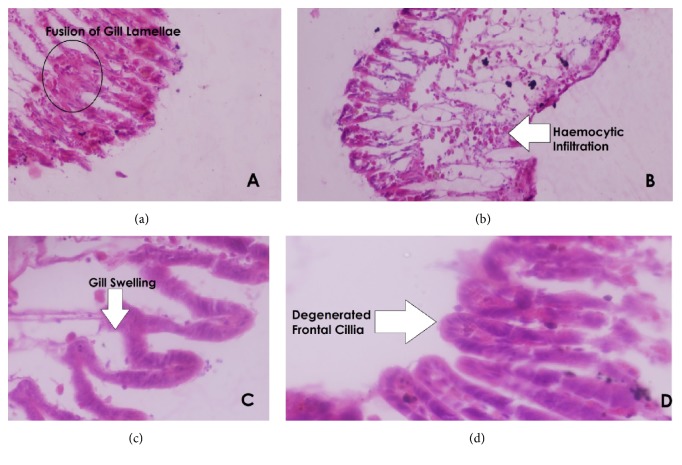
Section of gills showing (a) fusion of gills lamellae (×400), (b) hemocytic infiltration (×400), (c) gills swelling (×400), and (d) degenerated frontal gills (×400).

Histopathological alterations found in gills were lamellar fusion, dilated hemolymphatic sinus, degeneration of cilia, and hemocytic infiltration. Amangarh showed the highest degeneration of cilia, while Pir Sabaq manifests higher value of hemocytic infiltration. Samples collected from Amangarh had higher percentage (85%) of degeneration of cilia in gills (Figure 5).

#### 3.5.3. Gonads

**Figure 6:**
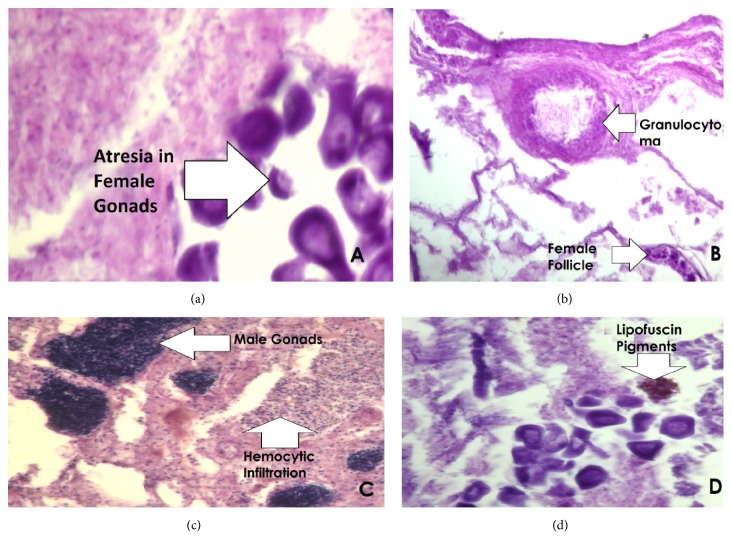
Section of gonads showing (a) atresia in female gonads (×400), (b) granulocytoma (×400), (c) hemocytic infiltration (×400), and (d) lipofuscin pigments (×400).

Histopathological alterations found were atresia, necrosis, granulocytoma, hemocytic infiltration, and lipofuscin pigments in gonads. Atresia (75%) was in highest prevalence in mussels from Sardaryab and Amangarh, while in the mussels samples from Michini Bridge, all other lesions were present except atresia (Figure 6).

Kabul River flowing through Pakistan has a number of biochemical characteristics. The dissolved oxygen levels within the river are generally good and above the usually recommended values, 5 gm/l, necessary for fisheries and aquatic life. BOD values within the river are generally acceptable. However, the downstream region is stressful for aquatic life and subject to organic pollution [51]. Now well established, most of the histopathological observations in an organism are related to environmental stresses [52]. Previous studies show increased mortality in freshwater mussels when exposed to metals [53]. Obviously other factors also play important role and one should also consider factor interaction.

The present study outlines major problems associated with heavy metal pollution in Kabul River. However, certain limitations are also linked to the study. Firstly, the river index should be calculated; secondly, the polycyclic aromatic hydrocarbons (PAHs) in the water, sediments, and mussel tissues should be estimated as they might contribute to the pollution and their consequences in the river fauna.

In Pakistan, few studies have been carried out to observe the effects of heavy metal pollution in aquatic or land ecosystems [54]. The consequences of heavy metal bioaccumulation and biomagnification on food chains should be further explored in the riverine habitats of the region. In this regard, present study lays the foundation for future research on local riverine ecology. Nevertheless, to enhance our knowledge of heavy metal pollution and its role in bioaccumulations and histopathological changes, further studies are quintessential.

## 4. Conclusion

In conclusion, the results indicate high concentrations of Cd, Cr, Cu, Fe, Mn Ni, Pb, and Zn in Kabul River. The study highlights the capability of heavy metals for bioaccumulation in the tissues of freshwater mussels and reveals histopathological alterations in mussel tissues. The study elaborates on vulnerability of riverine ecosystems to low levels of perturbation, that is, heavy metal pollution. It shows occurrence of bioaccumulation at permissible levels of pollutants. It further emphasizes the need of pollution mitigation activities in habitats of Kabul River. The results of this study are well under the scope and our main aims sand objectives of the study. The authorities should take necessary actions to save the river from pollution hazards.

## Figures and Tables

**Table 1:** Length, width, mussel weight, and mussel flesh (mean ± standard deviation) of *Anodonta cygnea.*

Site	Number	Length (mm)	Width (mm)	Mussel weight (g)	Mussel flesh (g)
Michini Bridge	20	113.7 ± 7.9	87.4 ± 6.3	239.6 ± 14.9	56.3 ± 5.6
Sardaryab	20	109.4 ± 4.5	92.3 ± 5.9	213.8 ± 8.4	63.2 ± 7.2
Amangarh	20	105.2 ± 9.1	72.1 ± 5.3	189.3 ± 11.8	39.2 ± 5.3
Pir Sabaq	20	87.9 ± 9.5	74.2 ± 9.3	145.9 ± 9.7	34.9 ± 5.9

**Table 2:** The standards used to analyze the variable using atomic absorption spectrophotometer (Spectra-AA-700).

Metals	Wavelength (nm)	Flame	Working range (*µ*g/mL)
Cd	228.8	AA (R)	0.5–2
Cr	357.9	AA (R)	2–8
Cu	324.8	AA (L)	2–8
Fe	279.5	AA (L)	1–4
Ni	324.7	AA (L)	3–12
Pb	283.3	AA (L)	5–20
Mn	248.3	AA (L)	1–4
Zn	213.9	AA (L)	0.4–1

AA, air acetylene; R, fuel-rich; L, fuel-lean.

**Table 3:** Mean concentration of metals (*µ*g/l) in water from Michini Bridge below Warsak Dam (reference site/site 1), Sardaryab, Amangarh, and Pir Sabaq (polluted sites 2, 3, and 4) of River Kabul receiving industrial effluents and city sewages and permissible limits of these metals according to NEQS.

Metals (*µ*g/l)	Site 1	Site 2	Site 3	Site 4	NEQS (*µ*g/L)
Cd	7.0 ± 4.0	82.3 ± 1.1^*∗∗*^	27.0 ± 1.7^*∗*^	90.6 ± 3.7^*∗∗*^	100
Cr	36.3 ± 4.9	51.0 ± 2.0^*∗*^	56.0 ± 4.3^*∗*^	64.0 ± 1.0^*∗∗*^	1000
Cu	24.3 ± 9.0	28.0 ± 1.1^*∗*^	27.6 ± 1.1^*∗*^	32.0 ± 3.0^*∗*^	1000
Fe	25.0 ± 5.0	53.0 ± 2.0^*∗∗*^	56.0 ± 1.5^*∗∗*^	63.6 ± 6.1^*∗∗*^	8000
Ni	68.0 ± 2.6	71.0 ± 1.0	76.0 ± 1.0	78.3 ± 0.5^*∗*^	1000
Pb	32.0 ± 6.0	170.3 ± 5.0^*∗∗*^	182.0 ± 5.29^*∗∗*^	186.3 ± 6.1^*∗∗*^	500
Mn	29.0 ± 5.0	56.0 ± 0.5^*∗∗*^	61.0 ± 2.0^*∗∗*^	64.0 ± 1.0^*∗∗*^	1500
Zn	104.3 ± 4.0	237.3 ± 5.1^*∗∗*^	243.3 ± 3.7^*∗∗*^	247.3 ± 2.0^*∗∗*^	500

Data is shown as Mean ± SD. NEQS, National Environmental Quality Standards. Difference is significant relative to site 1 at ^*∗*^*P* < 0.01 and ^*∗∗*^*P* < 0.001.

**Table 4:** Mean concentration of metals (mg/Kg) in sediments from Michini Bridge below Warsak Dam (reference site/site 1), Sardaryab, Amangarh, and Pir Sabaq (polluted sites 2, 3, and 4) of River Kabul receiving industrial effluents and city sewages.

Metals (mg/kg)	Site 1	Site 2	Site 3	Site 4
Cd	4.4 ± 2.6	5.0 ± 1.3^*∗*^	4.9 ± 1.7^*∗*^	7.1 ± 1.8^*∗*^
Cr	75.5 ± 24.8	85.4 ± 15.4	92.0 ± 10.2	92.5 ± 12.8
Cu	10.9 ± 2.9	14.2 ± 6.9	14.1 ± 6.5	15.3 ± 8.8
Fe	270.0 ± 97.9	344.6 ± 115.6^*∗*^	382.6 ± 43.3^*∗∗*^	591.3 ± 153.5^*∗*^
Ni	69.1 ± 18.8	85.1 ± 3.6^*∗∗*^	68.5 ± 13.3	82.6 ± 17.6^*∗*^
Pb	32.8 ± 18.2	40.3 ± 15.0^*∗*^	44.0 ± 9.7^*∗*^	54.6 ± 4.3^*∗∗*^
Mn	73.2 ± 24.8	78.3 ± 20.4	80.26 ± 8.1	83.7 ± 6.9^*∗∗*^
Zn	85.9 ± 10.5	86.4 ± 7.0^*∗*^	81.3 ± 16.8	88.3 ± 7.9^*∗∗*^

Data is shown as Mean ± SD. Difference is significant relative to site 1 at ^*∗*^*P* < 0.05 and ^*∗∗*^*P* < 0.001.

**Table 5:** Mean concentration of metals (*µ*g/g wet weight) in soft tissues of freshwater mussels collected from Michini Bridge below Warsak Dam (reference site/site 1), Sardaryab, Amangarh, and Pir Sabaq (polluted sites 2, 3, and 4) of River Kabul receiving industrial effluents and city sewages.

Metals (mg/kg)	Site 1	Site 2	Site 3	Site 4
Cd	0.9 ± 0.8	1.2 ± 0.7	1.2 ± 0.9	1.0 ± 0.6
Cr	1.1 ± 0.9	1.5 ± 0.7	1.7 ± 0.9	1.8 ± 0.9^*∗*^
Cu	2.7 ± 1.3	3.6 ± 0.6	3.2 ± 0.9	3.3 ± 1.0
Fe	76.3 ± 31.6	101.0 ± 30.3	162.3 ± 90.2^*∗∗∗*^	136.2 ± 62.3^*∗∗*^
Ni	1.5 ± 0.9	2.3 ± 1.0	1.6 ± 1.1	2.7 ± 4.7
Pb	10.1 ± 10.7	26.5 ± 11.2^*∗∗∗*^	26.1 ± 7.3^*∗∗∗*^	31.9 ± 11.9^*∗∗∗*^
Mn	35.1 ± 12.7	38.2 ± 13.5	39.9 ± 13.7	45.3 ± 19.5^*∗*^
Zn	41.7 ± 16.6	46.1 ± 13.7	47.4 ± 13.2	50.4 ± 13.7^*∗*^

Difference is significant relative to site 1 at  ^*∗*^*P* < 0.05,^*∗∗*^*P* < 0.01, and ^*∗∗∗*^*P* < 0.001.
